# Local Transmission of Imported Endemic Syphilis, Canada, 2011

**DOI:** 10.3201/eid1806.111421

**Published:** 2012-06

**Authors:** Sergio Fanella, Kamran Kadkhoda, Michelle Shuel, Raymond Tsang

**Affiliations:** University of Manitoba, Winnipeg, Manitoba, Canada (S. Fanella);; Cadham Provincial Laboratory, Winnipeg (K. Kadkhoda);; National Microbiology Laboratory, Winnipeg, (M. Shuel, R. Tsang)

**Keywords:** Treponema pallidum subsp. endemicum, bacteria, endemic treponematoses, RFLP, tpr genes, syphilis, transmission, Canada

## Abstract

Endemic (nonvenereal) syphilis is relatively common in nonindustrialized regions of the world. We describe a case of local transmission in Canada and review tools available for confirming a diagnosis. Improved molecular tools and global clinical awareness are needed to recognize cases of endemic syphilis imported to areas where it is not normally seen.

*Treponema pallidum* subsp. *endemicum* is the causative agent of endemic syphilis, also called nonvenereal syphilis. Other diseases caused by nonvenereal treponematoses are yaws (*T. pallidum* subsp. *pertenue*) and pinta (*T. carateum*). The 3 diseases are a substantial cause of illness in the nonindustrialized world, but they are rarely encountered in industrialized areas. Endemic syphilis is encountered in dry, hot regions, including Sahelian areas of western Africa and parts of Botswana, Zimbabwe, and the Arabian Peninsula (*1**–**4*). The causative organism is transmitted by direct contact with secretions from lesions or on fomites. The clinical spectrum of these diseases involves various degrees of involvement of the skin, mucous membranes, and skeletal system, depending on the organism (*1**,**2**,**5**,**6*).

## The Study

In February 2011, a 1-year-old girl was referred to our infectious diseases clinic for assessment of skin lesions. The patient had a history of several flesh-colored papules on her forehead at 2 months of age and on other areas of her body (hands, wrists, axilla, and anus) over the next several months. One week before our assessment, she began a 5-day course of azithromycin. Improvement in the lesions and resolution of an oral ulcer were reported by the parents at the assessment. The child appeared well. On examination in our clinic, flesh-colored papules were found on her right hand, wrist, and right axilla. A 1-cm condylomatous perianal lesion was also seen. The remainder of her examination findings were normal. Serologic screening for antibodies to *T. pallidum* was performed by using the Venereal Disease Research Laboratory (VDRL) test, which resulted in a 1:32 dilution, and the *T. pallidum* particle agglutination (TP-PA) assay of 4+ reactivity. Long bone radiographic results were normal.

The patient was born in Winnipeg, Canada, to a 39-year-old woman after a non-eventful pregnancy. The mother’s antenatal serologic results were negative for HIV and hepatitis B, and for syphilis by negative VDRL and TP-PA test results. The family lived in a refugee camp in the Republic of Senegal for 20 years before immigrating to Canada in November 2009. Review of laboratory records and discussions with public health services revealed that most of the family members had VDRL and TP-PA tests at a community clinic in early 2010 for unknown reasons, but they were lost to follow-up. The clinic was permanently closed and records were not available. The family had not traveled nor received visitors from overseas since moving to Canada.

We assessed all family members. Results of their examination and testing are described in Table 1. The patient’s 3-year-old brother had a 2-month history of progressive drooling and a hoarse voice. On examination, a 2 cm–diameter ulcer with raised edges and friable appearance was noted on the inside of his lower lip. He had a hoarse cry when agitated and drooled excessively, and he had palpable bilateral cervical lymphadenopathy. The remainder of his physical examination showed no abnormalities. His VDRL test result was positive at a 1:32 serum dilution, as was TP-PA with 4+ reactivity. PCR was performed on swab specimens from oral lesions by using previously described protocols (*7*) to detect the presence of 3 *T. pallidum* genes: *bmp*, *polA*, and *tpp47*. All 3 genes were detected.

**Table 1:** Serologic testing results for *Treponema pallidum* subspecies *endemicum* and clinical findings for a symptomatic patient and family members, Canada, 2011*

Family member†	Age, y/sex	VDRL result	TPPA result	Clinical findings	Molecular detection/PCR
Parent	40/F	Negative	Negative	None	ND
Parent	49/M	Negative	ND	None	ND
Child	1/F	1:32	4+‡	Skin papules, perianal condylomata	–§
Child	3/M	1:32	4+	Oral ulcer, hoarse voice, drooling, adenopathy	+¶
Child	5/F	Weakly reactive	4+	None	ND
Child	7/F	1:2	4+	None	ND
Child	10/F	1:8	4+	None	ND
Child	11/M	1:4	4+	None	ND
Child	14/M	1:16	4+	None	ND
Child	16/F	Weakly reactive	4+	None	ND
Child	19/F	1:16	4+	None	ND

*VDRL, Venereal Disease Research Laboratory test; TPPA, *T. pallidum* particle agglutination assay; ND, not done. †All family members received intramuscular benzathine penicillin (1.2 million U if >10 y old, 0.6 million U if <10 y old) after confirmatory PCR results for the 3-year-old child. ‡Positive reactions were measured on a scale of 1–4, with 4 as the highest value. §Oral swab specimen. Child had history of oral ulcer but had received a full course of azithromycin followed by oral lesion resolution before PCR was performed. She was born in Canada, had no travel history, and although the PCR result was negative, the test was performed after the patient received treatment. The patient had positive serologic test results for *T. pallidum*, a history of lesions characteristic of endemic syphilis, and no other plausible explanation for findings. ¶Oral ulcer swab specimen.

To determine whether the 3-year-old boy was infected with venereal syphilis or for nonvenereal *T. pallidum*, PCR was performed on a sample. The acidic repeat protein (*arp*) gene was amplified from the clinical specimen by using PCR primers and conditions as described (*8*). DNA sequencing of the purified PCR amplicons showed that the *arp* gene contained a central region of eight 60-bp repeats; all repeats contained identical nucleotide sequences (GenBank accession no. JN674561). The translated amino acid sequence, REVEDVPKVVEPASEREGGE, is characterized as a Type II repeat motif and has been described only in *arp* genes from nonvenereal *T. pallidum* subspecies (*8**,**9*).

Additional differentiation was accomplished by identification of subspecies-specific signature sequences in the *tprI* and *tprC* genes (*10*): the genes were amplified from the clinical specimen and from syphilis control DNA prepared from *T. pallidum* subsp. *pallidum* Nichols strain by using PCR. Because DNA for *T. pallidum* subsp. *endemicum* and *T. pallidum* subsp. *pertenue* was not commercially available, it was not included in this study. The subspecies-specific signatures were identified by restriction fragment length polymorphism (*10*) and DNA sequencing.

To characterize the *tprI* gene, PCR amplicons were digested with the restriction enzyme *Bsr*DI (New England Biolabs, Pickering, Ontario, Canada). The *tprI* gene from the clinical specimen was digested into 2 fragments of 334 bp and 159 bp (Figure), whereas *tprI* amplicons from the syphilis control strain were not digested and remained as a single band of 493 bp. DNA sequencing of the *tprI* gene from the clinical specimen confirmed the presence of a *Bsr*DI site (NN/CATTGC at position 1759–1766), which is typical for *T. pallidum* subsp. *endemicum*; no restriction site was observed in the syphilis control strain or in GenBank sequences for *T. pallidum* subsp. *pertenue* (Table 2).

**Figure:**
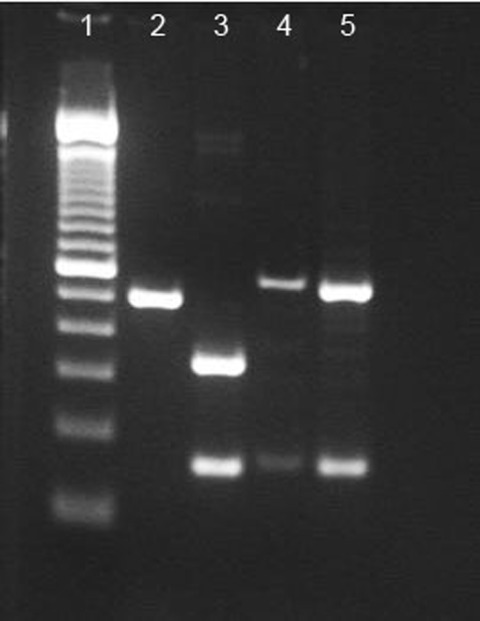
*Bsr*DI digest of *tprI* and *Bsr*DI/*Bs*EI double digest of *tprC* from the oral ulcer swab specimen and the syphilis control strain Nichols. Lanes from left to right: 1, 100-bp ladder; 2, Nichols *tprI* product not digested with BsrDI (493-bp band); 3, clinical specimen *tprI* product digested with *Bsr*DI (334-bp and 159-bp bands); 4, Nichols *tprC* digested with *Bsr*DI/*Bsi*EI (547-bp and 160-bp bands); 5, clinical specimen *tprC* digested with *Bsr*DI/*Bsi*EI (547-bp and 160-bp bands).

**Table 2:** Comparison of nucleotide sequences of *tprI* and *tprC* genes detected in clinical specimens from cases of endemic syphilis, Canada, 2011*

Strain†	*tprI* gene sequence at nt positions 1740–1766, 5′ → 3′	GenBank accession no.
Clinical strain	**CT**C **C**GA **T**G**T** TCC **C**TA CAT GGG **C**AT TGC	JN674562
Bosnia A	**CT**C **C**GA **T**G**T** TCC **C**TA CAT GGG **C**AT TGC	DQ886678
Nichols‡	**TG**C **T**GA **C**G**C** TCC **T**TA CAT GGG **T**AT TGC	NC_000919
Samoa D	**TG**C **T**GA **C**G**C** TCC **T**TA CAT GGG **T**AT TGC	DQ886680
	*tprC* gene sequence at nt positions 1704–1736, 5′ → 3′	
Clinical strain	**G**GT GCT **C**TC **C**GA **T**G**T** TCC **C**TA CAT GGG **C**AT T**G**C	JN674563
Bosnia A	**G**GT GCT **C**TC **C**GA **T**G**T** TCC **C**TA CAT GGG **C**AT T**G**C	DQ886673
Nichols‡	**C**GT GCT **T**GC **T**GA **C**G**C** TCC **T**TA CAT GGG **C**AT T**G**C	NC_000919
Samoa D	**G**GT GCT **C**TC **C**GA **T**G**T** TCC **C**TA CAT GGG **T**AT T**A**C	DQ886671

*Boldface indicates differences in nucleotide sequences. *Bsr*DI restriction sites are underlined. †Sequences were obtained from GenBank: Bosnia A (*T. pallidum* subsp. *endemicum*), Samoa D (*T. pallidum* subsp. *pertenue*) and syphilis control strain Nichols (*T. pallidum* subsp. *pallidum*). ‡Sequence also confirmed in this study.

To characterize the *tprC* genes, we simultaneously digested PCR amplicons by using restriction enzymes *Bsr*DI and *Bsi*EI (New England Biolabs). Digestion of DNA from *T. pallidum* subsp. *endemicum* strains with *Bsr*DI yielded 2 bands, 547 bp and 160 bp. The syphilis control strain also showed bands at 547 bp and 160 bp after digestion with *Bsr*DI, because of a site found in only one *T. pallidum* subsp. *pallidum* strain (Figure). The restriction fragment length polymorphism patterns for the *tprC* genes from the clinical specimen and the syphilis control strain were identical (Figure). However, DNA sequencing of the *tprC* gene from the clinical specimen revealed a >99% match to *tprC* GenBank sequences from other *T. pallidum* subsp. *endemicum* strains, a ≈97% match to *T. pallidum* subsp. *pallidum* Nichols strain (the syphilis control strain), and a ≈98% match to GenBank sequences for *T. pallidum* subsp. *pertenue* (Table 2). Furthermore, DNA sequence confirmed the presence of a *Bsr*DI restriction site in the *tprC* gene, which would be absent in subspecies *pertenue*. Characterization of the *arp*, *tprI,* and *tprC* genes identified the etiology of these clinical cases as *T. pallidum* subspecies *endemicum*.

The boy was administered 600,000 IU of penicillin intramuscularly; 4 weeks later, the ulcer and hoarseness had resolved, and the boy drooled only occasionally. All household members were subsequently treated with penicillin intramuscularly (Table 1).

## Conclusions

We suspect the 1-year-old child acquired her infection through close contact with her 3-year-old brother and other siblings, who themselves acquired endemic syphilis while in the Republic of Senegal. The use of molecular techniques greatly assisted in confirming the clinical diagnosis.

Control programs of the World Health Organization and the United Nations Children’s Fund using long-acting penicillin helped reduce the incidence of endemic syphilis and yaws by ≈95% during the 1950s and 1960s. Unfortunately, changes in the administration and delivery of these programs led to an increase in the prevalence of nonvenereal treponematoses during the 1970s (*2**,**5**,**6*). A risk remains for importation of disease into areas where it is not endemic and, subsequently, for local transmission of the etiologic agent.

Diagnosis of imported cases and cases resulting from local transmission is confounded by a lack of experienced clinicians in the diagnosis of nonvenereal treponematoses, and the inability of serologic methods to differentiate these disease entities from venereal syphilis and from each other. Definitive diagnosis is also hampered by widespread unavailability of molecular diagnostics. This deficit necessitates an integrated effort to offer a reproducible reference service to all care providers in a timely and reliable manner to ensure the best clinical outcome, as well as appropriate follow-up for infection control and public health purposes.
